# Effect of Continuous Supportive Telephone Counselling on Improving Breastfeeding Self-efficacy in Mothers with Late Preterm Infants Four Months After Discharge: a Randomized, Controlled Study

**DOI:** 10.34763/jmotherandchild.20212501.d-20-00017

**Published:** 2021-10-11

**Authors:** Marzieh Mohammadian, Azam Maleki, Gholamreza Badfar

**Affiliations:** 1 Counselling in Midwifery, Department of Midwifery, School of Nursing and Midwifery, Zanjan University of Medical Sciences, Zanjan, Iran; 2 Maternal and Child Health, Social Determinants of Health Research Center, Zanjan University of Medical Sciences, Zanjan, Iran; 3 Gholamreza Badfar, Pediatrics, Department of Pediatrics, Faculty of Medicine, Abuzar Children’s Hospital, Ahvaz Jundishapur University of Medical Sciences, Ahvaz, Iran

**Keywords:** Telephone counselling, breastfeeding self-efficacy, preterm infant, supportive care, women health

## Abstract

**Background:**

Breastfeeding self-efficacy is an important motivational factor in the continuity of lactation in mothers with preterm infants.

**Objective:**

The study aimed to determine the effect of continuous supportive telephone counselling on improving breastfeeding self-efficacy in mothers with late preterm infants.

**Material and methods:**

This randomized, controlled study was carried out with 65 eligible mothers (control n = 32, intervention n = 33) recruited in Ahvaz, Iran, in 2020. The eligible women were allocated into two groups— intervention and control—according to the block design. Data were measured monthly up to four months after discharge using the Dennis Breastfeeding Self-Efficacy Questionnaire. The control group received only routine care. Continuous supportive telephone counselling was provided for the intervention group members daily for 14 days after neonatal discharge. Data were analyzed using chi-square, repeated measures analysis of variance, independent t-test, and paired t-test at the significant level of 0.05. Statistical analysis was performed using the SPSS 16.0 software (SPSS Inc., Chicago, IL, USA).

**Results:**

The overall score in breastfeeding self-efficacy showed a statistically significant difference between the two study groups during the four months after discharge compared to the pre-intervention stage (P = 0.001). In the intervention group, the mean score of breastfeeding self-efficacy increased from 33.18 to 53.48, and in the control group it decreased from 31.17 to 28.56.

**Conclusion:**

The results showed that continuous supportive telephone counselling can improve breastfeeding self-efficacy in mothers with preterm infants. The approach seems to be an acceptable basis for designing intervention programs in this field.

## Introduction

The development of any society depends on the health of its people, especially the health of infants and children who are the future makers of a country. Unfortunately, despite all efforts to prevent premature births and the birth of infants with low birth weights, the global birth rate of such infants is still high [1]. In Iran, the prevalence of premature births varies in different cities. According to the findings of the review article, the overall prevalence of premature birth in Iran is 9.2%. Tehran, with 30.4%, has the highest percentage, and Kermanshah, with 2%, has the lowest percentage [1]. Preterm infants are at risk for hypoglycemia, jaundice, nutritional problems, and respiratory distress due to reduced general growth and insufficient maturity [2]. Due to their physiological characteristics, these infants need special care to achieve normal development and survival [3].

Due to the importance of breast milk in the development of preterm infants, it is necessary to focus on promoting exclusive breastfeeding in these infants [4]. For this purpose, in most preterm infants, milk feeding is performed several times a day by gavage. As the baby grows, direct breastfeeding gradually replaces gavage. The transition period from feeding by gavage to the breast or bottle is variable for each infant, with no fixed protocol [5]. Despite regional and global measures to increase breastfeeding choices and extend the duration of exclusive breastfeeding, many mothers still report problems in the postpartum period that lead them to abandon exclusive breastfeeding [6]. This increases the risk of weight loss and stunted growth in early infancy [7]. Behavioural intention and choice of breastfeeding are influenced by some socioeconomic and demographic factors. Also, self-efficacy has been mentioned as one of the effective factors affecting the continuation of breastfeeding in the first six months after discharge [8].

The term *self-efficacy*, introduced by Bandura, means the belief and confidence in one’s ability to perform healthy behaviour [9]. In this regard, Nichols believes that the higher the breastfeeding self-efficacy of mothers, the longer the duration of exclusive breastfeeding [10]. Implementing interventions that emphasize breastfeeding training can help improve self-efficacy and other consequences of breastfeeding in preterm infants. In most of these studies, the training has been done face-to-face and to a limited extent by telephone [11,12]. There is an information gap on the effectiveness of continuous supportive telephone counselling on postoperative breastfeeding self-efficacy in women with preterm infants. Midwives are in a great position to provide maternal and child health services, so they can play a key role in supporting these mothers as counsellors. Considering the importance of breastfeeding in preterm infants, the role of self-efficacy in continuing breastfeeding in the post-discharge period, and the unavailability of a similar study, the present study aimed to determine the effect of continuous supportive telephone counselling on breastfeeding self-efficacy in mothers with late preterm infants.

## Material and methods

### Study aim and design

This study was a randomized, controlled study to determine the effect of continuous supportive telephone counselling on breastfeeding self-efficacy on 65 women with preterm infants in Ahvaz (Iran) in 2020.

### Setting

The research setting included the neonatal intensive care units of Sina, Abuzar, and Imam Khomeini hospitals in Ahvaz (Iran).

### Participants

The study population included mothers with late preterm infants who were hospitalized in these wards. We estimated sample size on the basis of previous studies, having pretest and post-test scores for the same outcome measure (breastfeeding self-efficacy scale) [4], with considering mean ± standard deviation in the control group (39.70 ± 12.21), in the intervention group (65.81 ±7.37), the power of 80%, and a 10% attrition rate, the sample size was determined as 33 people in each group.

Inclusion criteria were as follows: mothers with late preterm infants (34 to 37 weeks), hospitalization of the infant for at least 48 hours in the intensive care unit, ability to breastfeed at discharge (exclusive and partial breastfeeding), willingness to participate in the study, the infant was the result of a single pregnancy, no delay in intrauterine growth and no birth defects, and possession of a telephone number for counselling and telephone follow-up.

The participants were excluded from the study if they no longer wished to continue to cooperate, attended similar counselling sessions elsewhere, or had any contraindications to breastfeeding during the study.

### Procedure

**Figure 1:**
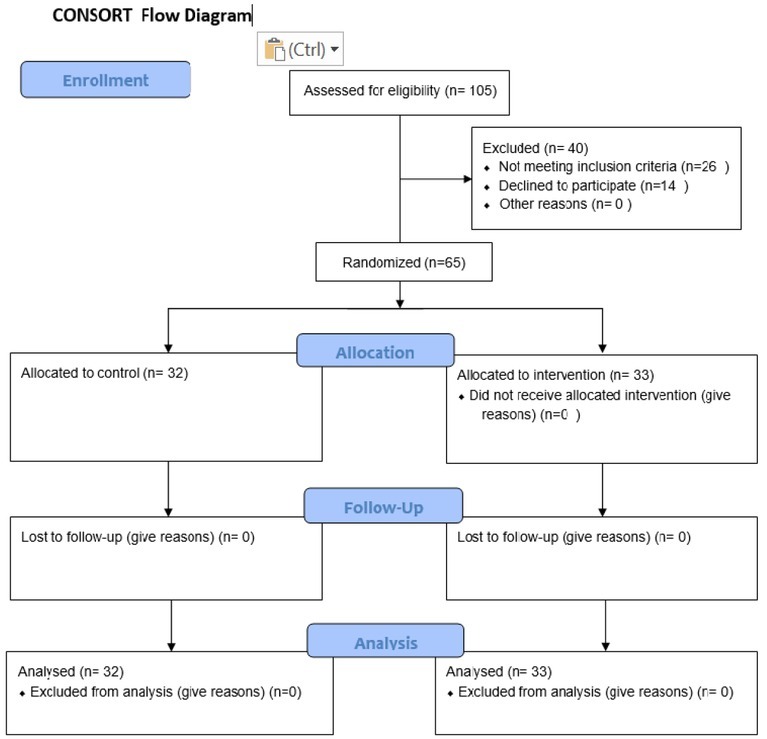
Flowchart of sampling and procedure

The convenience sampling method was employed to select eligible mothers, who were then allocated into two groups (intervention and control) according to the block design. In this way, six possible blocks were assigned to group A (intervention) and group B (control). Then, the number of blocks was selected from the random number table until the study sample size was reached. The size of the blocks was four. The whole four-patient block was placed in one envelope. To ensure the concealment of the sequence of enrolment, we used the sequentially numbered, opaque, sealed envelopes. The sequence of groups was provided by selecting each sheet after any referral to the centres for sampling; this method was repeated until the sample size was completed (control group n=32, intervention group n= 33) (Fig. 1).

### Intervention

The first two weeks after delivery are critical to improving breastfeeding self-efficacy. In the present study, therefore, the intervention group received 14 days of daily continuous supportive counselling by telephone after neonatal discharge. At discharge, the control group received routine care, including neonate screening tests and information about infant clinical signs to determine the need for readmission to the NICU. In Iran, similar to many other countries, breastfeeding education programs take place immediately after childbirth if the preterm infants don’t have contraindications to breastfeeding.

The content of the sessions was designed according to the latest protocols of the Ministry of Health under the supervision and advice of a neonatologist. Table 1 describes the intervention. The first session of counselling was face-to-face, and the remaining sessions were consistently held by telephone. The telephone counselling sessions were scheduled with maximum flexibility for the participants. According to the specified schedule, participants were contacted daily. It was also a two-way call, and mothers were reminded that they could call at any time of the day if necessary.

**Table 1:** The content of the counselling in terms of each session

The first session was conducted face to face at discharge time, and the rest of the sessions were conducted by telephone daily for 14 days.	
**First session (face to face)**	Welcome Aims and objectives Pre-test with Dennis Breastfeeding Self-Efficacy Observing the infant sucking power (latch) and mother–infant relationship during lactation Discussing the concept of breastfeeding self-efficacy, factors affecting breastfeeding, and breastfeeding self-efficacy
**The second session**	Discussing the baby’s general condition, jaundice, and respiratory, urinary, and faecal pattern. Discussing how to determine the adequacy of breastfeeding, hours, and frequency of breastfeeding Regular hand washing and personal hygiene in baby care and breastfeeding Mentioning the warning signs and the need to go to the relevant specialists
**Third session**	Repeating the content of the second session Encourage skin-to-skin contact between mother and baby and kangaroo mother care as well as exercise to enhance sucking in preterm infants Non-nutritious sucking, how to feed the baby with breast milk
**Fourth session**	Discussing the women’s beliefs and attitudes about breastfeeding, the importance of breast milk in the growth, and development of premature babies Discussing the role of the mother’s effort and cooperation in improving the breastfeeding process
**Fifth session**	Strengthening mothers’ self-confidence, encouraging them to increase their individual skills in baby care, and receiving help from others Follow-up for routine neonatal care, neonatal screening
**Sixth session**	Repeating the content of the second to fifth sessions. Discussing how to determine the adequacy of breastfeeding, hours, and frequency of breastfeeding Encourage skin-to-skin contact between mother and baby and kangaroo mother care as well as exercise to enhance sucking in preterm infants Mentioning the warning signs and the need to go to the relevant specialists
**Seventh up to Fourteenth sessions**	Encouraging mothers to express their feelings, needs, and desires to counsellors and others Reviewing the second to sixth session topics Discussing the baby’s general condition, mothers’ self-confidence and coping with baby care Discussing the women’s beliefs and attitudes about breastfeeding, the importance of breast milk in the growth and development of premature babies Discussing factors affecting breastfeeding and breastfeeding self-efficacy The counselling was with maximum flexibility for the participants

### Data collection instruments

Data were collected by the self-report method using a checklist of demographic characteristics and the Breastfeeding Self-Efficacy Questionnaire.

The demographic checklist, which was completed by self-report and by reference to patients’ files, included age of woman, education of woman, occupation of wife and husband, place of residence, number of previous pregnancies, adequacy of monthly family income, sex of baby, gestational age of baby at birth, and type of delivery. The breastfeeding self-efficacy questionnaire was completed in the pre-consultation stage and monthly up to four months after discharge.

### Dennis Breastfeeding Self-Efficacy Questionnaire (13 items)

The BSES-Short Form (BSES-SF) contains 14 items and is scored on a 5-point Likert scale, with responses ranging from 1 to 5. As such, total scores can range from 14 to 70. The Persian form of the questionnaire was approved using 13 questions; as a result, total scores can range from 13 to 65. All the questions start with the phrase “I can always do it.” Responses for each question are assessed points from one, for “I’m not sure at all,” to five, for “I’m always or completely sure.” A high score thus indicates more self-efficacy or self-sufficiency in breastfeeding [13]. Psychometrics of the Persian version of the 13-item breastfeeding self-efficacy questionnaire in Iran had good validity and reliability [14].

## Data analysis

Data were analysed using SPSS software version 16. The normality of the data was assessed using the Kolmogorov-Smirnov test. The data had a normal distribution. Chi-square test and Fisher’s Exact Test were used to compare qualitative variables between the two groups, and independent t-test and paired t-test were used to compare quantitative variables. The repeated measurement ANOVA test was used to evaluate the effect of time and group. The significance level was 0.05.

## Results

### Demographic characteristics

The results showed that most of the participants in the intervention group had university educations (33.3%) and most in the control group had a diploma education (34.4%). Both groups were multi-parity (63.6% vs. 50 %), employed (97% vs. 93.8%), with self-employed spouse occupations (66.7% vs. 71.9%), and residents of the city (81.8 vs. 96.9%). The number of female infants (60.6%) was higher in the intervention group, and the number of male infants (56.3%) was higher in the control group. The type of caesarean delivery was higher than normal delivery in both groups (72.7% vs. 87.5%); family incomes in both groups were sufficient (60.6%vs. 59.4%). The comparison of demographic characteristics between the two groups was not statistically significant, so the two groups were homogeneous (p < 0.05). See Table 2.

**Table 2:** Comparison of the frequency distribution of individual-social characteristics of participants between the two groups Intervention and control

Variable		Intervention		Control		P-value*
		Percentage	Frequency	Percentage	Frequency	
Education	Illiterate bondage	15.2	5	21.9	7	0.244*
	Middle–high school	21.2	7	31.3	10	
	Diploma	30.3	10	34.4	11	
	University	33.3	11	12.5	4	
Female occupation	Housewife	97	32	93.8	30	0.613**
	Employed	3	1	6.3	2	
Spouse job	Free	66.7	22	71.9	23	0.649*
	Employee	33.3	11	28.1	9	
Residence	City	81.8	27	96.9	31	0.050*
	Village	18.2	6	3.1	1	
Gender of the baby	Girl	60.6	20	43.8	14	0.174*
	Boy	39.4	13	56.3	18	
Type of delivery	Normal	27.3	9	12.5	4	0.137*
	Cesarean section	72.7	24	87.5	28	
Monthly income	Less than enough	39.4	13	40.6	13	0. 919*
	It is enough	60.6	20	59.4	19	
Parity	Primiparous	34.4	12	50	16	
	Multiparous	63.6	21	50	16	
Sex of baby	Female	60.6	20	43.8	14	0.174*
	Male	39.4	13	56.3	18	

*Chi-square ** Fisher’s Exact Test

The mean age of participants in the intervention group was 30.39 ± 5.97, while in the control group it was 28.34 ± 4.78 years. The gestational age of infants at birth in the intervention group was 34.93 ± 0.87, while in the control group it was 35.18± 0.84 weeks. The observed differences between the two groups in terms of participants’ age and neonatal age were not statistically significant (p <0.05).

## Breastfeeding self-efficacy

The results independent t-test showed that the mean score of breastfeeding self-efficacy before intervention was 33.18 ± 4.63 in the intervention group and 31.71 ± 4.28 in the control group. The comparison of the two groups’ mean scores of breastfeeding self-efficacy in the pre-intervention stage was not statistically significant (P =0.479). The mean score of breastfeeding self-efficacy in the control group increased at the first month of the follow-up period (32.59 ± 7.36), but it decreased from the second to the fourth month of the follow-up period compared to the period before the intervention. In the intervention group in the same time period, the mean breastfeeding self-efficacy increased from 33.18 to 53.48. The observed differences between the two groups were statistically significant (p = 0.001). The result of paired t-test showed that the comparison of the pre-test mean difference of breastfeeding self-efficacy between four point of post-test were statistically significant in the intervention group but not in the control group (Table 3).

**Table 3:** Comparison of breastfeeding self-efficacy scores between intervention and control groups in five time periods (before intervention and monthly up to 4 months after delivery

Variable	Time	Control	Intervention	P-value*
		Mean±SD	Mean±SD	
Breastfeeding self-efficacy	Before	31.71±4.28	33.18±4.63	0.479
	The first month	32.59±7.36	44.69±9.51	0.001
	The second month	32.75±8.86	48.45±9.26	0.001
	Third month	30.56±9.86	51.18±10.8	0.001
	The fourth month	28.56 ±10.71	53.48±10.13	0.001
	Paired t-test (P-value)	0.129**	0.001**	-

* Independent t-test, **Paired t-test

**Figure 2:**
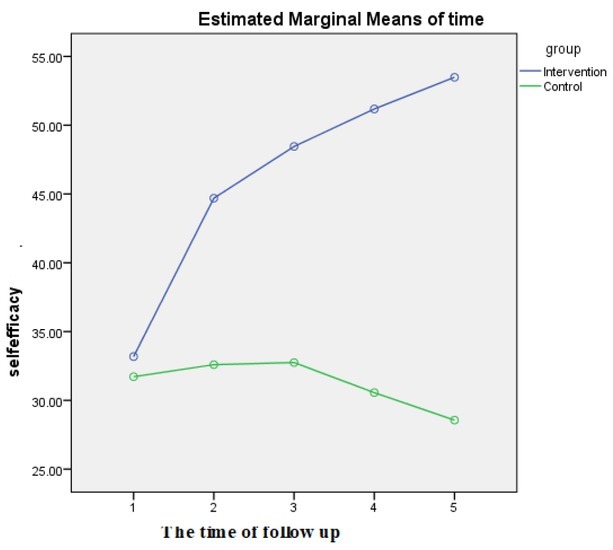
The variation of the self-efficacy score in five time periods

The result of the repeated measurement ANOVA test showed that the change of the mean scores in four time periods between the two groups was also significant (Fig. 2).

## Discussion

The results showed that continuous supportive telephone counselling could effectively improve breastfeeding self-efficacy in the intervention group. The mean breastfeeding self-efficacy in the intervention group increased from 33.18 to 53.48, whereas in the control group, it decreased from 31.17 to 28.56. Sibel Küçükoğlu et al. (2014) showed that performing five days of face-to-face breastfeeding counselling in the hospital and a phone call to mothers with preterm infants one week after discharge could increase breastfeeding self-efficacy up to six months after delivery. This study showed that mothers with higher self-efficacy had fewer breastfeeding problems [4], which is consistent with the results of the present study. It also shows that telephone follow-up after discharge can be effective in improving and maintaining breastfeeding self-efficacy. Brockway (2018) suggested that family-centred counselling could improve breastfeeding self-efficacy in mothers with preterm infants [15], which is consistent with the results of the present study. The current results also showed that telephone counselling, as a cost-effective method, can be as effective as direct counselling in improving breastfeeding self-efficacy in mothers of preterm infants. However, the effectiveness of telephone counselling other than for self-efficacy of breastfeeding in initiating or continuing breastfeeding in these infants has been emphasized in some studies, including the study of Ericson et al. (2018) and Lavender et al. (2013) [11,16]. Also in the study of Akbarian et al. (2017), telephone counselling was associated with a decrease in readmission of preterm infants after discharge [17]. In another study in 2020, Jang et al. reported similar results, showing that conducting three face-to-face training sessions during hospitalization and continuing telephone support counselling every 15 days until 6 months postpartum improved parenting confidence but not the exclusive breastfeeding rate in mothers with preterm infants [18]. It seems that breastfeeding difficulties in late-preterm infants can decrease mothers’ confidence. Bostanabad et al. reported similar results in 2019 [19]. The results of the above studies emphasized the importance of telephone counselling in the post-discharge period in improving neonatal-related outcomes that can be considered by health planners and policymakers. In a contradictory study, Chan Man et al. (2016) showed that having a face-to-face breastfeeding training session in the third trimester of pregnancy and a telephone counselling session in the first week after delivery had short-term effects on improving breastfeeding self-efficacy. The first week after delivery had a significant increase but was not effective in self-efficacy improvement after four weeks of delivery [12]. The results of the above study are not consistent with the results of the present study, probably due to the difference in the type of intervention method. In the present study, continuous supportive counselling was performed in the first two weeks after discharge. Breastfeeding self-efficacy means a person’s belief and confidence in her ability to perform healthy and breastfeeding behaviours [13], which is influenced by some individual and social factors. Breastfeeding self-efficacy in mothers with preterm infants and low birth weight is lower than that of other mothers [8,13]. Expanding the maternal support system, especially after discharge, can be considered one effective solution in this field. The important point about breastfeeding these infants is the presence of a caregiver who can encourage mothers to continue breastfeeding and be responsible for the mothers’ educational needs before and after the birth of the infant and can follow up after discharge [20]. According to the evidence, these mothers are often unable to attend face-to-face counselling classes after discharge due to their baby’s condition. Because software, messengers, and telephone calls are convenient, available, interactive, and reliable, they can be considered in the post-discharge service package for preterm infants [21]. However, due to the variety of protocols and possible costs for purchasing or installing software and using social networks, it is suggested that the effectiveness and cost-effectiveness of using online methods with a continuous consultation approach be compared with periodic consulting in future studies. According to the findings of the present study, it can be concluded that supportive telephone counselling was effective in improving breastfeeding self-efficacy in mothers with preterm infants. It seems that the present approach is an acceptable basis for designing intervention programs in this field, especially for mothers with middle to lower socioeconomic status.

All authors declare that our organizational affiliations are not dependent on any organizations sanctioned by the United States. The research centre is not dependent on sanctioned organizations.

### Limitations of the study

The present study encountered some limitations. For instance, the data were collected using self-reporting; blinding was not performed; and the cost-effectiveness of telephone calls was not investigated. Therefore, the results should be generalized according to these limitations. The continuity and level of breastfeeding, not calculated in this study, should be considered by researchers in future studies.

### Key points

- Breastfeeding self-efficacy is an important motivational factor at the beginning and for continuity of lactation in mothers with preterm infants.
- Continuous supportive telephone counselling can improve breastfeeding self-efficacy in mothers with preterm infants.
